# Travel-Associated Melioidosis in Non-Endemic Regions: A Systematic Review and Meta-Analysis

**DOI:** 10.3390/ijerph23010036

**Published:** 2025-12-25

**Authors:** Jongkonnee Thanasai, Atthaphong Phongphithakchai, Moragot Chatatikun, Sa-ngob Laklaeng, Jitbanjong Tangpong, Pakpoom Wongyikul, Phichayut Phinyo, Supphachoke Khemla, Anchalee Chittamma, Wiyada Kwanhian Klangbud

**Affiliations:** 1 Faculty of Medicine, Mahasarakham University, Mahasarakham 44000, Thailand; jongkonnee@msu.ac.th; 2 Nephrology Unit, Division of Internal Medicine, Faculty of Medicine, Prince of Songkla University, Songkhla 90110, Thailand; atthaphong.p@psu.ac.th; 3 School of Allied Health Sciences, Walailak University, Nakhon Si Thammarat 80160, Thailand; moragot.ch@wu.ac.th (M.C.); sumoun2528@gmail.com (S.-n.L.); rjitbanj@wu.ac.th (J.T.); 4 Research Excellence Center for Innovation and Health Products (RECIHP), Walailak University, Nakhon Si Thammarat 80160, Thailand; 5 Center for Clinical Epidemiology and Clinical Statistics, Faculty of Medicine, Chiang Mai University, Chiang Mai 50200, Thailand; aumkidify@gmail.com (P.W.); phichayutphinyo@gmail.com (P.P.); 6 Department of Biomedical Informatics and Clinical Epidemiology (BioCE), Faculty of Medicine, Chiang Mai University, Chiang Mai 50200, Thailand; 7 Division of Infectious Diseases, Department of Internal Medicine, Nakhon Phanom Hospital, Nakhon Phanom 48000, Thailand; sup.mednkp@gmail.com; 8 Department of Pathology, Faculty of Medicine Ramathibodi Hospital, Mahidol University, Bangkok 10400, Thailand; anchalee.chi@mahidol.ac.th; 9 Medical Technology Program, Faculty of Science, Nakhon Phanom University, Nakhon Phanom 48000, Thailand

**Keywords:** melioidosis, *Burkholderia pseudomallei*, imported infection, travel medicine, global health, sepsis

## Abstract

**Background**: Travel-associated melioidosis, caused by *Burkholderia pseudomallei*, is increasingly reported in non-endemic countries due to rising global travel. Understanding demographic, clinical, and outcome patterns of imported cases is important to improve recognition and management in settings where melioidosis is uncommon. **Methods**: We systematically searched PubMed, Embase, and Scopus (last search: 24 September 2025) for case reports and case series of melioidosis diagnosed outside endemic regions and linked to travel exposure. Data were extracted on demographics, comorbidities, clinical manifestations, and outcomes. We performed descriptive analyses, subgroup analyses, and Firth’s penalized logistic regression to explore predictors of death. The protocol was registered in PROSPERO (CRD420251154559). **Results**: A total of 104 studies, encompassing 143 individual cases, were included. Most diagnoses occurred in non-endemic, high-income countries, especially the Netherlands (21%), France (10%), the United States (9%), and South Korea (7%). Infections were predominantly acquired in Southeast Asia, particularly Thailand (39%). The mean patient age was 50.6 years, with a male predominance (78%). Diabetes mellitus was the most frequent comorbidity (28%). Clinical presentations included pulmonary (33%), sepsis (27%), cutaneous (13%), abdominal (4%), and osteoarticular disease (1%). Overall mortality was 12.6% and relapse occurred in 7%. In penalized regression analyses, no baseline characteristic was statistically significantly associated with mortality; septic presentation showed an elevated point estimate for odds of death, but with imprecise estimates. **Conclusions**: Travel-associated melioidosis is a rare but clinically significant imported infection. Most cases followed exposure in Southeast Asia, and pulmonary disease and sepsis were the most frequent presentations. Mortality remained substantial (12.6%), and relapse was reported in 7%, underscoring the need for early recognition, appropriate therapy, and follow-up in non-endemic settings.

## 1. Introduction

Melioidosis is a life-threatening infectious disease caused by the Gram-negative bacillus *Burkholderia pseudomallei*, naturally found in soil and water in tropical regions, particularly Southeast Asia and northern Australia. It has been estimated to cause around 165,000 cases and 89,000 deaths globally each year, underscoring its considerable public health burden [1,2]. Despite the availability of effective antimicrobials such as ceftazidime and meropenem, mortality remains unacceptably high, often surpassing 40% in septicemic presentations [3,4]. Diabetes mellitus is the most important risk factor, with chronic kidney disease, alcohol use, and immunosuppression also conferring susceptibility [5,6]. With increasing globalization, international travel and migration have brought melioidosis into non-endemic regions, where low clinical suspicion and limited laboratory capacity contribute to delayed diagnosis and inappropriate treatment [7,8]. Although the overwhelming burden of melioidosis occurs in endemic regions, imported cases in travelers and migrants are particularly relevant to non-endemic health systems because they are prone to delayed recognition, laboratory misidentification, and severe outcomes despite access to care; they also serve as sentinels of geographic spread and travel-related exposure risks. Moreover, treatment is complicated by the risk of relapse, which has long been recognized when eradication therapy is inadequate [9], and recent systematic reviews highlight variability in eradication regimens, particularly with co-trimoxazole, that continue to affect outcomes [10].

Although the clinical and epidemiological importance of imported melioidosis has been increasingly recognized, the available literature remains fragmented. For instance, a European review highlighted diagnostic challenges and frequent misdiagnoses but was geographically restricted [5]. Similarly, a South Korean case series demonstrated that all patients had Southeast Asian exposure and a 36% fatality rate, yet the analysis was limited to a small cohort [11]. More recently, a narrative global review identified 137 travel-associated cases, describing diabetes and pneumonia as dominant features, but this work remained descriptive and did not employ systematic or quantitative approaches to estimate pooled outcomes [12]. Risk factor analyses from endemic regions further emphasize that mortality is closely tied to comorbidities such as chronic kidney disease, hypoalbuminemia, and uncontrolled diabetes [13], but the extent to which these predictors apply to imported cases remains unclear. Collectively, these studies highlight the relevance of imported melioidosis but demonstrate a lack of systematically synthesized, quantitative evidence that could inform risk stratification, relapse prevention, and mortality predictors across non-endemic countries.

The present systematic review and meta-analysis addresses this gap by consolidating individual patient data from published case reports and case series of travel-associated melioidosis diagnosed in non-endemic regions, where melioidosis is uncommon and diagnostic delays are more likely. We acknowledge that the greatest burden of melioidosis remains in endemic settings; however, imported cases represent a distinct clinical and public health challenge because delayed recognition, limited laboratory familiarity, and differences in empiric management pathways may substantially influence outcomes in non-endemic healthcare systems. Our objectives are to describe the global distribution of imported cases by both country of diagnosis and country of exposure, to summarize patient demographics and underlying risk factors, to characterize the spectrum of clinical manifestations and outcomes (including relapse), and to explore predictors of mortality through pooled analyses. By integrating scattered data into a comprehensive synthesis, this work provides a quantitative summary of travel-associated melioidosis to support improved clinical suspicion and earlier diagnosis in returning travelers, inform treatment and relapse prevention strategies, and strengthen travel medicine and surveillance efforts in the context of increasing international mobility.

## 2. Materials and Methods

### 2.1. Protocol and Registration

The systematic review and meta-analysis were conducted in accordance with the Preferred Reporting Items for Systematic Reviews and Meta-Analyses (PRISMA 2020) guidelines [14]. The review protocol was prospectively registered in the International Prospective Register of Systematic Reviews (PROSPERO) under the registration number CRD420251154559. The protocol outlined the research question, eligibility criteria, data extraction process, and planned methods for qualitative synthesis and meta-analysis.

### 2.2. Search Strategies

A comprehensive search of PubMed, Embase, and Scopus was performed on 24 September 2025. Detailed search strategies for each database are provided in Supplementary Table S1. No restrictions on language or publication date were applied. Additional records were identified through manual reference screening.

### 2.3. Eligibility Criteria

We included case reports and case series describing human melioidosis acquired during international travel or in non-endemic regions following exposure abroad. Studies were excluded if they (i) described animal infections, (ii) were reviews or conference abstracts without original data, or (iii) did not provide sufficient clinical or outcome information for extraction.

We operationally defined melioidosis-endemic regions as geographic areas with established autochthonous melioidosis transmission and/or environmental suitability with documented presence of *Burkholderia pseudomallei*, as summarized in major reviews and global distribution maps [3]. Specifically, we considered Southeast Asia (e.g., Thailand, Malaysia, Singapore, Vietnam, Cambodia, Laos, Myanmar, the Philippines, and Indonesia), northern Australia, Papua New Guinea, South Asia (e.g., India, Bangladesh, Sri Lanka, and Nepal), and southern China (including Hainan) and Taiwan as endemic/suspected-endemic areas. Non-endemic regions were defined as countries outside these areas where locally acquired cases are not established; thus, included cases were diagnosed in non-endemic countries with travel exposure in endemic or suspected-endemic locations.

### 2.4. Data Extraction

From each eligible case, we extracted author/year, country of diagnosis, country of origin of infection, demographic variables (age, sex), risk factors (e.g., diabetes mellitus), clinical manifestations, and outcomes (death, relapse). Clinical manifestations were grouped into sepsis, pulmonary, cutaneous, osteoarticular, abdominal, and other categories for standardized analysis.

### 2.5. Risk of Bias Assessment

As all included reports were single-patient case reports, the risk of bias was inherently high. We did not formally apply case-report quality appraisal tools, but data completeness and consistency were assessed.

### 2.6. Statistical Analysis

Descriptive statistics were used to summarize demographics, clinical features, and outcomes. Continuous variables were reported as mean ± SD and categorical variables as frequencies (%). Associations between risk factors and mortality were tested using Fisher’s exact or chi-square tests. To evaluate predictors of death, we fit Firth’s penalized likelihood logistic regression to address sparse data and quasi-complete separation. Results are presented as odds ratios (ORs) with 95% confidence intervals (CIs), and statistical significance was assessed at a two-sided α = 0.05. Analyses were conducted using R version 4.4.

## 3. Results

### 3.1. Included Studies

**Figure 1:**
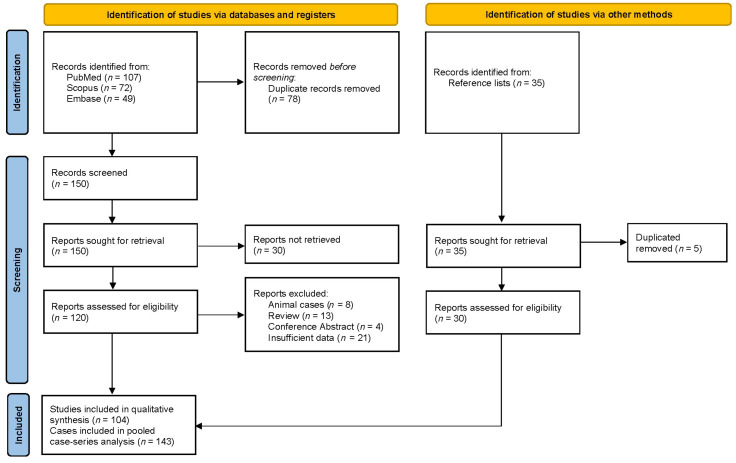
PRISMA flow diagram of study selection.

The database search yielded a total of 228 records (PubMed, *n* = 107; Scopus, *n* = 72; Embase, *n* = 49), and an additional 35 records were identified through reference lists. After the removal of 83 duplicates (78 and 5), 180 unique records were screened. During the screening process, 30 records were excluded due to ineligibility, most commonly because they did not report travel-associated/imported human melioidosis diagnosed in non-endemic settings (e.g., endemic-country cohorts without travel linkage), were non-original publications (reviews/editorials), or lacked extractable individual patient data. The full text of 150 articles was then assessed for eligibility, of which 46 were excluded (animal cases, *n* = 8; reviews, *n* = 13; conference abstracts, *n* = 4; insufficient or insufficient data, *n* = 21). In addition, 35 records were identified from reference lists. Then, 5 duplicated records were removed. Ultimately, 104 studies were included in the qualitative synthesis, contributing a total of 143 travel-associated melioidosis cases to the pooled case-series analysis. The study selection process is illustrated in Figure 1 (PRISMA flow diagram).

### 3.2. Study Characteristics

A total of 143 travel-associated melioidosis cases were identified from 104 published case reports and case series [11,15,16,17,18,19,20,21,22,23,24,25,26,27,28,29,30,31,32,33,34,35,36,37,38,39,40,41,42,43,44,45,46,47,48,49,50,51,52,53,54,55,56,57,58,59,60,61,62,63,64,65,66,67,68,69,70,71,72,73,74,75,76,77,78,79,80,81,82,83,84,85,86,87,88,89,90,91,92,93,94,95,96,97,98,99,100,101,102,103,104,105,106,107,108,109,110,111,112,113,114,115,116,117]. These reports originated mainly from non-endemic, high-income countries, reflecting the global spread of imported melioidosis. The Netherlands (*n* = 30, 21%), France (*n* = 14, 10%), the United States (*n* = 13, 9%), and South Korea (*n* = 10, 7%) accounted for the majority of diagnoses, with additional reports from Denmark, the United Kingdom, Germany, Finland, Australia, and China. This distribution underscores the burden of imported melioidosis in regions where clinical suspicion may be low. The details were shown in Table 1. The details were in Supplementary File S1.

### 3.3. Origin of Infection

Most cases were linked to travel in Southeast Asia, particularly Thailand (*n* = 56, 39%), followed by Malaysia, Vietnam, the Philippines, India, Indonesia, Bangladesh, Cambodia, and Myanmar. This geographical pattern mirrors the well-known endemic distribution of *Burkholderia pseudomallei*, highlighting the risk to returning travelers from the region. As shown in Table 1.

### 3.4. Demographic Characteristics

The mean age of patients was 50.6 years (SD 16.3; range 6–90 years). The age distribution indicated clustering around the fifth and sixth decades of life, although both children and older adults were represented. There was a strong male predominance (78.3%, *n* = 112) compared with females (21.7%, *n* = 31). This gender pattern is consistent with endemic cohorts and may reflect occupational and behavioral exposure factors. As shown in Table 1.

### 3.5. Risk Factors

Diabetes mellitus was the most frequently reported comorbidity, present in 28% (*n* = 40) of cases, as shown in Table 1. Other risk factors, such as alcohol use, chronic kidney disease, and immunosuppression, were inconsistently reported and thus could not be analyzed systematically (Supplementary File S1). The predominance of diabetes among cases is consistent with melioidosis epidemiology in endemic settings.

### 3.6. Clinical Manifestations

Clinical presentations were heterogeneous but could be categorized into six main groups. Pulmonary involvement was most frequent, observed in 47 cases (32.9%), followed by sepsis (*n* = 39, 27.3%). Cutaneous manifestations accounted for 18 cases (12.6%), while abdominal abscesses were observed in five cases (3.5%), and osteoarticular infections in two cases (1.4%). An additional 32 cases (22.4%) presented with other or unspecified forms. These findings illustrate the broad clinical spectrum of melioidosis in travelers, with both localized and disseminated forms documented. As shown in Table 1.

### 3.7. Clinical Outcomes

The overall mortality rate was 12.6% (*n* = 18/143), and relapse occurred in 7% (*n* = 10/143) of patients. Mortality among patients with sepsis was higher (23.1%) compared with those with non-sepsis presentations (8.7%), though this difference was not statistically significant (*p* = 0.37), as shown in Table 2.

### 3.8. Subgroup Analyses

Mortality did not significantly differ between patients with and without diabetes (15% vs. 12%; *p* = 0.79). Similarly, there was no significant difference in mortality by sex, with rates of 13.3% among men and 9.7% among women (*p* = 0.76). Although sepsis carried the highest case fatality rate, the association did not reach statistical significance, likely due to the small number of fatal cases. Data were shown in Table 2.

### 3.9. Regression Analyses

Due to sparse data and quasi-complete separation, we used Firth’s penalized logistic regression to obtain bias-reduced estimates of mortality predictors. No baseline characteristics reached statistical significance; sepsis had the highest point estimate for mortality (OR 3.81, 95% CI 0.37–519.0), with wide uncertainty. Age, sex, and diabetes mellitus were not associated with death (Table 3).

## 4. Discussion

This systematic review provides the most comprehensive synthesis to date of travel-associated melioidosis, consolidating data from 143 cases reported in 104 studies [11,15,16,17,18,19,20,21,22,23,24,25,26,27,28,29,30,31,32,33,34,35,36,37,38,39,40,41,42,43,44,45,46,47,48,49,50,51,52,53,54,55,56,57,58,59,60,61,62,63,64,65,66,67,68,69,70,71,72,73,74,75,76,77,78,79,80,81,82,83,84,85,86,87,88,89,90,91,92,93,94,95,96,97,98,99,100,101,102,103,104,105,106,107,108,109,110,111,112,113,114,115,116,117] across multiple decades and continents. Several important findings emerge, with significant implications for both clinical practice and global health.

The majority of diagnoses were reported from non-endemic, high-income countries in Europe, North America, and East Asia. This pattern reflects both the rising volume of international travel and the heightened awareness and diagnostic capability in high-resource settings [118]. Thailand emerged as the most frequent country of exposure, consistent with its high endemic burden of *Burkholderia pseudomallei* [119], coupled with its popularity as a global tourist destination [120]. These findings align with prior epidemiological estimates that identify Southeast Asia as the epicenter of melioidosis risk [121]. Notably, the diversity of exposure locations—including Malaysia, Vietnam, India, and Indonesia—emphasizes that imported melioidosis is not confined to one nation but represents a regional hazard across tropical Asia.

The demographic characteristics of imported cases mirrored those of endemic cohorts. The predominance of middle-aged males likely reflects occupational exposures (such as agriculture or outdoor activities) as well as behavioral risk factors [8]. Importantly, diabetes mellitus was present in over one-quarter of patients, reaffirming its role as the single strongest host risk factor for melioidosis [8]. The pathophysiological basis for this association lies in impaired neutrophil chemotaxis, phagocytosis, and oxidative burst among diabetic patients [122], all of which contribute to increased susceptibility to *B. pseudomallei*. Other comorbidities such as alcohol misuse, chronic kidney disease, and immunosuppression were occasionally reported, but inconsistently extracted in the literature, limiting quantitative analysis.

Imported melioidosis presented with a wide clinical spectrum. Pulmonary infection and septicemia were the dominant manifestations, consistent with endemic patterns, while cutaneous, abdominal, and osteoarticular forms were less common. The heterogeneity of presentations underscores the challenge for clinicians in non-endemic regions, where diagnostic suspicion is often low, and misdiagnosis as tuberculosis or bacterial pneumonia is frequent [25,41,83,85,92].

In Firth’s penalized regression, no baseline predictors of death reached statistical significance; however, sepsis had the highest point estimate for mortality with very wide confidence intervals, reflecting limited power in case-report data. Clinically, this supports maintaining a high index of suspicion and initiating appropriate empiric therapy (e.g., ceftazidime or meropenem) and critical care support when severe sepsis is suspected. Notably, the overall mortality of imported cases (12.6%) was lower than often reported in endemic cohorts (40–50%), which may relate to earlier access to intensive care and effective antimicrobials in high-resource settings.

Relapse was observed in 7% of cases [25,51,74,95,101], consistent with the well-documented propensity of *B. pseudomallei* to persist intracellularly and recrudesce following incomplete eradication. Importantly, relapse rates may be underreported in case reports and short series, as long-term follow-up is frequently unavailable. This highlights the need for adherence to prolonged eradication therapy with oral agents such as trimethoprim-sulfamethoxazole and the establishment of follow-up protocols even in non-endemic countries.

Previous narrative reviews have emphasized the rarity but clinical severity of travel-associated melioidosis [12]. However, none have pooled data systematically across such a large number of individual patients. Our review extends existing knowledge by quantifying risk factors, clinical outcomes, and predictors of mortality using standardized analytic approaches. Importantly, our findings corroborate endemic studies while contextualizing the distinct challenges of diagnosis and management in non-endemic settings.

This review has several strengths. It is the largest synthesis of imported melioidosis to date, incorporates cases from a broad geographic distribution, and applies both descriptive and regression analyses. The use of PRISMA methodology and a registered PROSPERO protocol enhances transparency and reproducibility.

Nonetheless, limitations must be acknowledged. The reliance on case reports and small case series introduces publication bias, as unusual or severe cases are more likely to be published. Clinical data were often incomplete or heterogeneously reported, limiting subgroup analyses. The relatively small number of deaths restricted the statistical power of regression models, producing wide confidence intervals and non-significant associations for some predictors. Finally, long-term outcomes such as relapse are likely underestimated due to a lack of follow-up reporting in the case-based literature.

Our findings have practical implications. Clinicians in non-endemic countries should maintain a high index of suspicion for melioidosis in febrile travelers returning from Southeast Asia, particularly those with pneumonia or sepsis and underlying diabetes. Diagnostic laboratories outside endemic regions should be equipped to correctly identify *B. pseudomallei*, as misidentification as Pseudomonas species remains common. Public health authorities should consider travel health advisories for at-risk groups, particularly diabetic travelers engaging in activities involving soil or water exposure.

Future research should aim to strengthen global surveillance systems for imported melioidosis, expand access to point-of-care diagnostics in non-endemic regions, and develop preventive strategies for high-risk travelers. Collaborative international registries may help overcome the limitations of case report–based evidence and provide more robust epidemiological insights.

## 5. Conclusions

Travel-associated melioidosis is a rare but clinically significant imported infection diagnosed in non-endemic regions. Most cases followed exposure in Southeast Asia, particularly Thailand, and the most frequent clinical presentations were pulmonary disease (33%) and sepsis (27%). Overall mortality was 12.6% and relapse was reported at 7%. In Firth’s penalized regression, no baseline characteristics were statistically significant predictors of death, although sepsis had the highest point estimate with wide uncertainty. Clinicians in non-endemic countries should consider melioidosis in returning travelers with pneumonia or systemic infection and ensure appropriate eradication therapy and follow-up to reduce relapse.

Future work should prioritize standardized reporting of travel-associated cases (including treatment regimens and long-term follow-up), and develop international registries to better quantify relapse and identify prognostic factors for severe outcomes in non-endemic settings.

## Figures and Tables

**Table 1:** Characteristics of the 104 included studies reporting 143 travel-associated melioidosis cases.

Category	Sub-Category/Country	*n* (%) or Description
**Study Characteristics**	Study type—Case reports	97 (93.3%)
	Study type—Case series	7 (6.7%)
	Publication years	1977–2024
	1977–1999	18
	2000–2010	32
	2011–2020	47
	2021–2024	7
	Patients per study	Median 1 (range 1–3)
**Country of Diagnosis**	Netherlands	30 (21%)
	France	14 (10%)
	United States	13 (9%)
	South Korea	10 (7%)
	Denmark	8 (6%)
	United Kingdom	8 (6%)
	Germany	7 (5%)
	Finland	5 (3%)
	Australia	4 (3%)
	China	3 (2%)
	Others	41 (28%)
**Origin of Infection**	Thailand	56 (39%)
	Malaysia	8 (6%)
	Vietnam	7 (5%)
	Philippines	4 (3%)
	India	4 (3%)
	Indonesia	4 (3%)
	Bangladesh	4 (3%)
	Cambodia	4 (3%)
	Myanmar	4 (3%)
	Other: Southeast Asia	12 (8%)
	Others (by region)	36 (25%) (Africa, Americas/Caribbean, Oceania, and Middle East; see Supplementary File S1 for full details)
**Demographics**	Mean age	50.6 ± 16.3 (range 6–90)
	Male	112 (78.3%)
	Female	31 (21.7%)
**Risk factors**	Diabetes mellitus	40 (28%)
**Clinical manifestations**	Pulmonary	47 (32.9%)
	Sepsis	39 (27.3%)
	Cutaneous	18 (12.6%)
	Abdominal	5 (3.5%)
	Osteoarticular	2 (1.4%)
	Other */unspecified	32 (22.4%)
**Outcomes**	Mortality	18 (12.6%)
	Relapse	10 (7.0%)

* Other: alcohol use, renal disease, immunosuppression, etc., inconsistently reported.

**Table 2:** Clinical outcomes and subgroup analyses.

Variable	Mortality *n*/*N* (%)	*p*-Value	Relapse *n*/*N* (%)
**Overall**	18/143 (12.6%)	–	10/143 (7.0%)
**Sex**			
Male	15/112 (13.3%)	0.76 †	–
Female	3/31 (9.7%)		–
**Diabetes mellitus**			
Yes	6/40 (15.0%)	0.79 ‡	–
No	12/103 (11.7%)		–
**Clinical presentation**			
Sepsis	9/39 (23.1%)	0.37 †	–
Non-sepsis	9/104 (8.7%)		–

† Fisher’s exact test; ‡ chi-squared test with Yates’ correction. Relapse subgroup analyses were not performed because follow-up and baseline characteristics were inconsistently reported among relapse cases.

**Table 3:** Firth’s penalized logistic regression for predictors of mortality.

Predictor	OR (95% CI)	*p*-Value
Age (per year)	0.997 (0.966–1.029)	0.85
Male sex	1.08 (0.32–4.54)	0.91
Diabetes mellitus	1.20 (0.40–3.35)	0.73
Sepsis (vs. other)	3.81 (0.37–519.0)	0.31

OR—odd ratio.

## Data Availability

No new data were created.

## Supplementary Materials

The following supporting information can be downloaded at https://www.mdpi.com/article/10.3390/ijerph23010036/s1: Supplementary Table S1. Search strategies for each database; Supplementary File S1. Characteristics of patients.

## Abbreviations

The following abbreviations are used in this manuscript:

CI	Confidential interval
OR	Odd ratio
SD	Standard deviation
