# Supplementary material for: Travel-Associated Melioidosis in Non-Endemic Regions: A Systematic Review and Meta-Analysis
Source: Int J Environ Res Public Health. 2025 Dec 25;23(1):36. doi: 10.3390/ijerph23010036 (PMC12840764; doi:10.3390/ijerph23010036)
Supplement: Supplementary file 1 [file ijerph-23-00036-s001.zip › Supplementary_Table_S1_Search_Strategies.pdf]

Global Burden and Clinical Outcomes of Travel-Associated Melioidosis: A Systematic Review and Meta-analysis of Imported cases in Non-Endemic Countries

Jongkonnee Thanasai[1], Atthaphong Phongphithakchai[2], Moragot Chatatikun[3,4], Sa-ngob Laklaeng[3], Jitabanjong Tangpong[3,4], Pakpoom Wongyikul[5,6], Phichayut Phinyo[5,6], Supphachoke Khemla[7], Anchalee Chittamma[8], Wiyada Kwanhian Klangbud[9,*]

[1] Faculty of Medicine, Mahasarakham University, Mahasarakham 44000, Thailand; jongkonnee@msu.ac.th (J.T.)
[2] Nephrology Unit, Division of Internal Medicine, Faculty of Medicine, Prince of Songkla University, Songkhla 90110, Thailand; atthaphong.p@psu.ac.th
[3] School of Allied Health Sciences, Walailak University, Nakhon Si Thammarat 80160, Thailand: moragot.ch@wu.ac.th (M.C.); sumoun2528@gmail.com (S.-n.L.); rjitbanj@wu.ac.th (J.T.)
[4] Research Excellence Center for Innovation and Health Products (RECIHP), Walailak University, Nakhon Si Thammarat 80160, Thailand; moragot.ch@wu.ac.th (M.C.); rjitbanj@wu.ac.th (J.T.)
[5] Center for Clinical Epidemiology and Clinical Statistics, Faculty of Medicine, Chiang Mai University, Chiang Mai 50200, Thailand; aumkidify@gmail.com (P.W.); phichayutphinyo@gmail.com (P.P.)
[6] Department of Biomedical Informatics and Clinical Epidemiology (BioCE), Faculty of Medicine, Chiang Mai University, Chiang Mai 50200, Thailand; aumkidify@gmail.com (P.W.); phichayutphinyo@gmail.com (P.P.)
[7] Division of Infectious Diseases, Department of Internal Medicine, Nakhon Phanom Hospital, Nakhon Phanom 48000, Thailand; sup.mednkp@gmail.com
[8] Department of Pathology, Faculty of Medicine Ramathibodi Hospital, Mahidol University, Bangkok 10400, Thailand; anchalee.chi@mahidol.ac.th
[9] Medical Technology Program, Faculty of Science, Nakhon Phanom University, Nakhon Phanom 48000, Thailand; wiyadakwanhian@gmail.com
* Correspondence: wiyadakwanhian@gmail.com

Supplementary Table S1. Search strategies for each database

Database	Search Query / Strategy	Results (n)
PubMed	("Melioidosis"[MeSH Terms] OR melioidosis OR "Burkholderia pseudomallei") AND ("travel" OR traveler* OR "imported infection" OR migration OR tourism)	107
Scopus	TITLE-ABS-KEY (melioidosis OR "Burkholderia pseudomallei") AND TITLE-ABS-KEY (travel OR traveler* OR "imported infection" OR migration OR tourism)	72
Embase	'melioidosis'/exp OR 'Burkholderia pseudomallei' AND (travel OR traveler* OR 'imported infection' OR migration OR tourism)	49
Manual reference screening	Reference lists of included articles	35
